# An adenine model of inborn metabolism errors alters TDP-43 aggregation and reduces its toxicity in yeast revealing insights into protein misfolding diseases

**DOI:** 10.15698/mic2025.05.850

**Published:** 2025-05-22

**Authors:** Sangeun Park, Sei-Kyoung Park, Peter Blair, Susan W. Liebman

**Affiliations:** 1 Department of Pharmacology, University of Nevada, Reno, United States of America.

**Keywords:** yeast, TDP-43, liquid-like droplets, metabolite-based amyloids, FRAP, metabolism disorders, ALS

## Abstract

TDP-43 is linked to human diseases such as amyotrophic lateral sclerosis (ALS) and frontotemporal degeneration (FTD). Expression of TDP-43 in yeast is known to be toxic, cause cells to elongate, form liquid-like aggregates, and inhibit autophagy and TOROID formation. Here, we used the *apt1*∆* aah1*∆ yeast model of inborn errors of metabolism, previously shown to lead to intracellular adenine accumulation and adenine amyloid-like fiber formation, to explore interactions with TDP-43. Results show that the double deletion shifts the TDP-43 aggregates from liquid-like droplets toward a more amyloid-like state. At the same time the deletions reduce TDP-43’s effects on toxicity, cell morphology, autophagy, and TOROID formation without affecting the level of TDP-43. This suggests that the liquid-like droplets rather than amyloid-like TDP-43 aggregates are responsible for the deleterious effects in yeast. How the *apt1*∆* aah1*∆ deletions alter TDP-43 aggregate formation is not clear. Possibly, it results from adenine and TDP-43 fiber interactions as seen for other heterologous fibers. This work offers new insights into the potential interactions between metabolite-based amyloids and pathological protein aggregates, with broad implications for understanding protein misfolding diseases.

## Abbreviations

FUS - fused in sarcoma,

SDS - sodium dodecyl sulfate,

TDP-43 - TAR DNA-binding protein 43,

TOROID - TORC1 organized in inhibited domain.

## INTRODUCTION

Certain proteins and peptides have been shown to be associated with disease when they form amyloid (or amyloid-like) oligomers or fibers. Amyloids are ordered protein aggregates characterized by a cross-β sheet structure that binds to thioflavin T 1. Such proteins include: A(, α-synuclein, TDP-43 (TAR DNA-binding protein 43), FUS (fused in sarcoma), Huntingtin, and p53 which each form aggregates associated, respectively, with Alzheimer’s, Parkinson’s, amyotrophic lateral sclerosis (ALS)/ frontotemporal degeneration (FTD), Huntington’s, and cancer 2,3,4. Many of these proteins can also form liquid-like droplets through liquid-liquid phase separation in the cytoplasm. These droplets can be dissolved by hexanediol, exhibit dynamic properties, and can convert into amyloid fibrils 5.

In most cases it is unclear which aggregate form is toxic. Indeed, the very structural nature of TDP-43 aggregates, whether found in patients, or formed *in vitro*, remains a subject of debate 6. Some studies suggest that the low-complexity C-terminal domain of TDP-43 forms amyloid *in vitro* 7, while others report that neither *in vitro*-formed nor neuron-based TDP-43 aggregates exhibit amyloid properties 6,8. Although TDP-43 aggregates are linked to disease, it is unclear whether they contribute to disease pathology or are merely a consequence of it.

Expression of TDP-43 in yeast causes toxicity 9 and a dramatic elongation of cell shape 10. Mutations in TDP-43 which either increase or decrease its toxicity in yeast, hint that amyloid aggregates may actually have a protective role. Toxicity-enhancing intragenic mutations reduced TDP-43 hydrophobicity, while toxicity-reducing mutations increased hydrophobicity and encouraged larger TDP-43 aggregates in the cytoplasm 11. However we found that expression of wild-type, non-toxic and toxicity-enhancing TDP-43 protein in yeast formed aggregates that were dissolved by hexanediol and were not stained with thioflavin T 10. This indicates that all TDP-43 forms liquid-like droplets and not amyloid in yeast.

Expression of FUS in yeast is also toxic. However, unlike TDP-43, FUS aggregates stain with thioflavin T and do not disappear when cells are treated with hexanediol 10,12,13,14.

Extragenic mutations also affect toxicity when TDP-43 is expressed in yeast 9,15,16,17,18,19,20. The mechanism by which TDP-43 causes toxicity remains unknown. However, we found that expression of wild-type TDP-43 in yeast reduces autophagy and TOROID (TORC1 organized in inhibited domain) formation. Extragenic and intragenic mutations which alleviate TDP-43 toxicity, reverse this effect 10,20. TOROIDs are large, helical structures of TORC1 that form near the vacuole and are associated with reduced autophagy inhibition.

Yeast prions also form amyloid. [*PSI*^+^] is the prion form of the translational release factor, SUP35. We showed that the *de novo* appearance of [*PSI*^+^] is dramatically enhanced by the presence of another yeast prion, [*PIN*^+^] (the prion form of RNQ1). Both the [*PSI*^+^] and [*PIN*^+^] prions can form different heritable amyloid shapes with distinct characteristics, called prion strains. Some [*PIN*^+^] strains destabilize [*PSI*^+^] strains and different [*PIN*^+^] strains preferentially promote the appearance of different [*PSI*^+^] strains. Other heterologous amyloid aggregates can also facilitate the *de novo* appearance of [*PSI*^+^] 21,22,23,24,25,26,27.

This type of interaction also occurs in mammalian cells. Aggregates of α-synuclein facilitate fibrillization of tau 28, and Aβ polymerization is promoted by misfolded type-2 diabetes islet amyloid polypeptide 29,30. This suggests that cross-seeding is a risk factor for disease.

Recently individual amino acids, and non-proteinaceous metabolites including adenine have also been shown to form amyloid-like fibers that bind thioflavin T and are toxic. Since inborn errors of metabolism disorders lead to accumulation of these fibers and cause neurological symptoms a "generic amyloid hypothesis" has been proposed to include both protein misfolding diseases and inborn errors of metabolism diseases 31,32,33.

Since these diseases are associated with an increased frequency of neurodegeneration and cancer, it is possible that the metabolite fibers serve as scaffolds upon which pathological protein aggregation can initiate 34.

A model for adenine self-assembly in living yeast was established by deleting two enzymes in the adenine salvage pathway: *APT1* (encoding adenine phosphoribosyltransferase) and *AAH1* (encoding adenosine deaminase). This double deletion causes a dramatic increase in the level of intracellular adenine which increases further when cells are grown in the presence of adenine. Furthermore, the increased intracellular adenine is amyloid-like and associated with growth inhibition 32.

Here, we use this yeast model to show that *apt1*Δ* aah1*Δ double deletions alter the properties of human TDP-43 expressed in yeast.

## RESULTS

### Examining the toxicity of TDP-43 in yeast in the presence of *apt1*Δ* aah1*Δ

**Figure 1:**
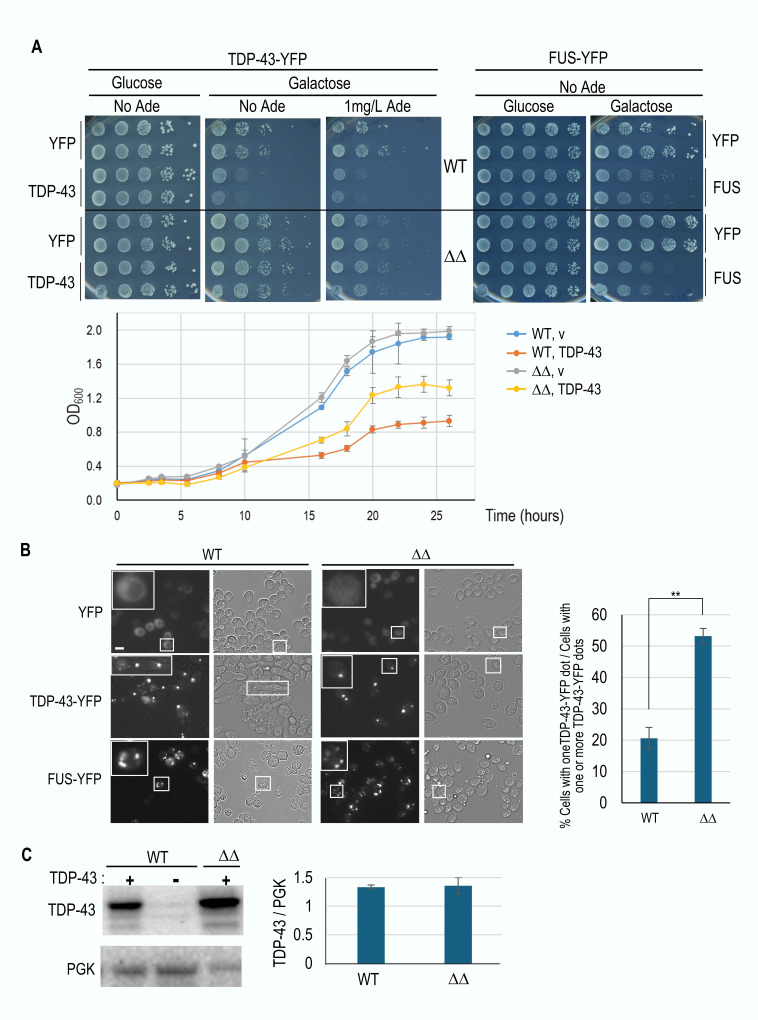
FIGURE 1: The *aah1*Δ* apt1*Δ deletions alleviate TDP-43 but not FUS toxicity. **(A)** The double *aah1*Δ *apt1*Δ deletion alleviated toxicity associated with expression of TDP-43. TDP-43-YFP or FUS-YFP were expressed from the *GAL *promoter on *CEN* plasmids (*YCpGAL-TDP43-YFP*, and *YCpGAL-FUS-YFP*) in wild-type BY4741 (WT) or an isogenic double* aah1*Δ *apt1*Δ deletion strain (ΔΔ). Normalized transformants were serially diluted, spotted on plates (upper) or grown in liquid (lower). Cells were grown in plasmid selective media with glucose (non-inducing) or galactose (inducing YFP, TDP-43-YFP or FUS-YFP) as indicated. YFP-expressing cells were used as controls (*YCpGAL-EYFP*). No relief of FUS toxicity was observed in the* aah1*Δ *apt1*Δ strain compared to the wild type. **(B)** The double *aah1*Δ *apt1*Δ deletion changes the appearance of TDP-43 foci and alleviates the elongated cell shape caused by expression of TDP-43. Cells expressing YFP, TDP-43-YFP, or FUS-YFP on selective galactose plates were collected and examined for YFP fluorescence (left) and bright-field microscopy (right). All images were taken at the same magnification. Scale bar is 10 µm. Magnified cells are boxed. Graph on right shows the percentage of cells containing a single TDP-43-YFP aggregate vs. total cells with one or more aggregates. Data are presented as the mean ± standard error of the mean (n=3). More than 500 cells were counted. Statistical significance was determined with a two-tailed t-test (** = p < 0.01). (C) The double deletion doesn’t reduce TDP-43 expression. Left shows a representative Western blot of the WT and double deletion (ΔΔ) strains with galactose induced TDP-43 or an empty vector with loading control, phosphoglycerate kinase (PGK). The lane in the middle is a control without TDP-43. The bar chart includes data from three independently made ΔΔ strains each run on three separate gels. Error bars represent the SEM.

As reported previously when TDP-43-YFP is expressed in yeast on galactose it forms foci, reduces growth rate, and causes an elongation of cell shape (**Fig. 1A, B**) 9,35. The double *apt1*Δ* aah1*Δ deletion (ΔΔ) significantly reduces this toxicity, resulting in a growth rate in between that seen with and without TDP-43 in wild-type cells (**Fig. 1A**). The ΔΔ mutant also restores cells to their normal shape and causes a significant reduction in the number of cells with a multiple vs single TDP-43-YFP foci per cell (**Fig. 1B**, see also **Fig. 4A**).

A reduction in TDP-43 toxicity is observed with or without the addition of adenine to the media (**Fig. 1A**) and this is not caused by a reduction in TDP-43 expression (**Fig. 1C**). Addition of adenine doesn’t affect the growth rate of wild-type cells but as reported previously 32 it reduces the growth rate of cells bearing the *apt1*Δ* aah1*Δ double deletion (**Fig. 1A**). The double deletion was specific for TDP-43, it didn’t alter the toxicity of FUS (**Fig. 1A**).

### Examining the effect of *apt1*Δ *aah1*Δ on inhibition of autophagy and TOROID formation caused by TDP-43 expression

**Figure 2:**
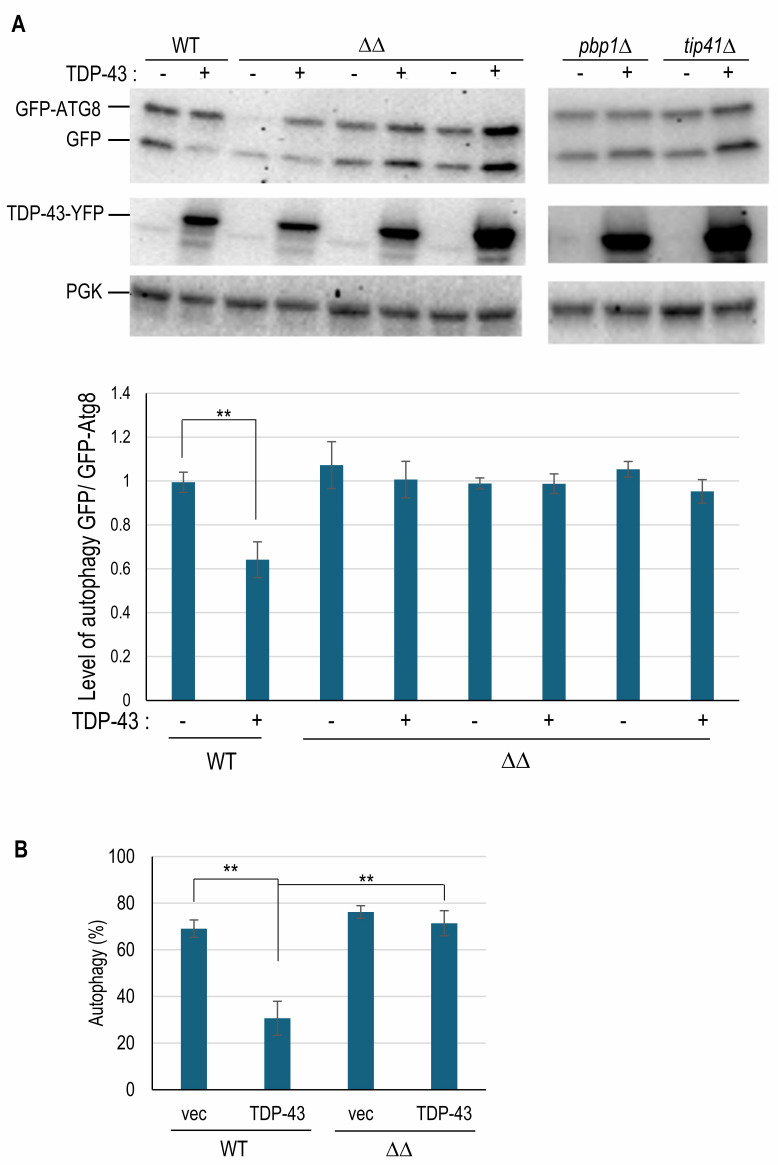
FIGURE 2: The *aah1*Δ* apt1*Δ double deletion reverses the inhibition of autophagy caused by overexpression TDP-43. **(A)** Autophagy measured by Western blots. Autophagy measured by the breakdown of GFP-ATG8 is shown on Western blots. BY4741 (WT) and three independently made isogenic double deletion strains (ΔDelta;) were transformed with *pGAL-TDP43-L* and p*CUP1-GFP-ATG8-U* and grown on 2% galactose plates with 1% raffinose, the required amino acids methionine and histidine, and 50 µM copper sulfate for 16 hours at 30^o^C before being harvested for Western blotting. One representative blot is shown on the top left. The bar chart shows data from three independent double deletion strains repeated three times each and from a wild-type control. Also included as a control is a gel showing the breakdown of GFP-ATG8 in cells with the TDP-43 modifiers *tip41*Δ and *pbp1*Δ (upper right)*. *As shown previously 20 these modifiers prevented TDP-43 from reducing autophagy. **(B)** Autophagy measured microscopically. Double transformants in BY4741 (WT) and isogenic double deletion strains (ΔΔ) shown in (A) were assayed for autophagy by determining the cellular location of GFP-ATG8 microscopically. Autophagy was measured as the fraction of cells with either no GFP-ATG8 fluorescence or with fluorescence only in the vacuole over total live cells. There was no significant difference in autophagy without TDP-43 expression (vec, vector) in the wild-type (WT) and double deletion strains (ΔΔ). Error bars represent the SEMs calculated from the average of three independent transformants by examining 250-550 cells per transformant. ** indicates p <0.01 in a paired two-tailed t-test.

The expression of wild-type TDP-43 in yeast reduces autophagy relative to the vector control 20. As in our previous papers, we measured autophagy by using immunoblotting to examine the breakdown of GFP-ATG8 (Green Fluorescent Protein fused to autophagy-related protein 8) releasing free GFP (**Fig. 2A**) and by examining the cellular location of GFP in cells expressing GFP-ATG8 (**Fig. 2B**) 10,20. We previously found that modifiers (mutations in *TIP41 *or *PBP1*) that reduce toxicity of TDP-43 also reduce TDP-43’s inhibition of autophagy 10,20. Similarly, our current results using both immunoblotting and cellular location measures of autophagy show that the level of autophagy is not altered by *apt1*Δ* aah1*Δ in the absence of TDP-43. However, both methods show that the presence of *apt1*Δ* aah1*Δ prevented overexpression of TDP-43 from inhibiting autophagy (**Fig. 2A, B**)*. *

**Figure 3:**
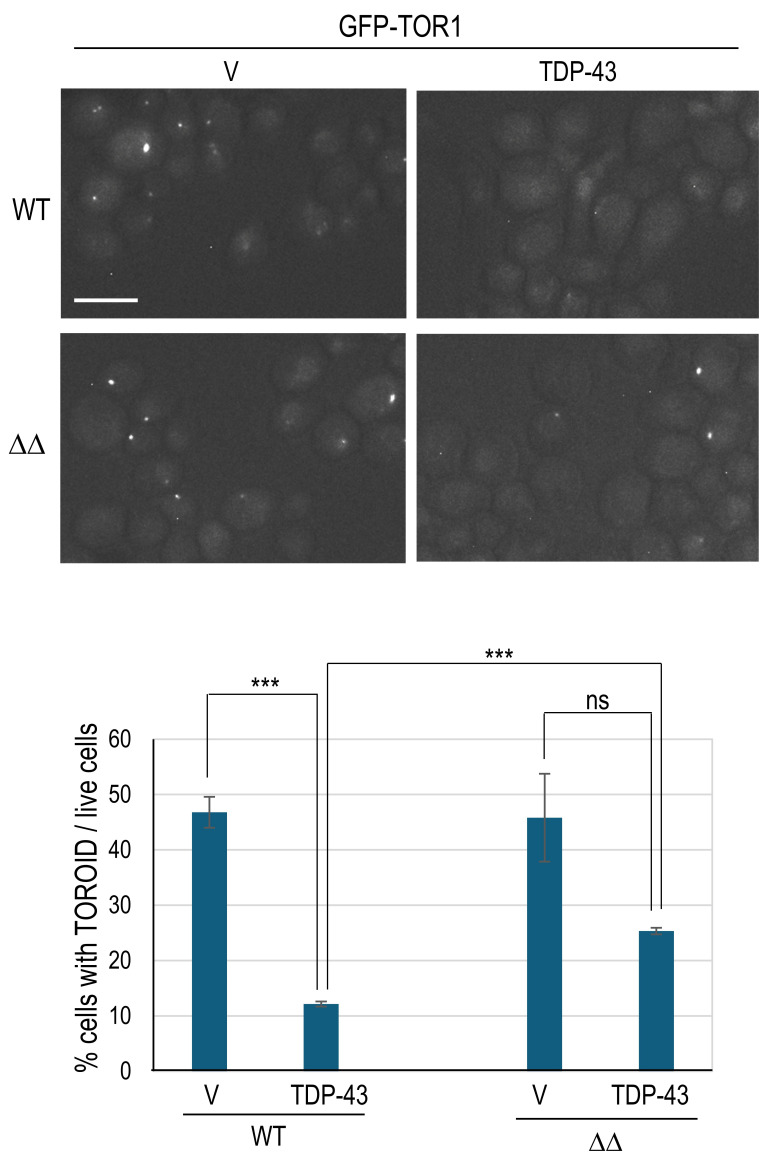
FIGURE 3: The *aah1*Δ* apt1*Δ double deletion reduces the inhibition of TOROID formation caused by TDP-43 overexpression. Shown are fluorescent images of endogenously tagged *TOR1* (*GFP-TOR1*) BY4741 cells with (ΔΔ) or without (WT) the double *aah1*Δ* apt1*Δ deletion. Cells were transformed with untagged TDP-43 expressed under the *GAL* promoter in *YCpGAL-TDP43-U *(TDP-43) or empty vector *YCpGAL-U* (v), grown on plasmid selective galactose media for 2 days, examined with a FITC filter and photographed (top). Scale bar is 10 µm. The percentage of cells with a GFP-TOR1 dot (TOROID) among live cells expressing TDP-43 or empty vector was counted after 0.5% trypan blue staining to exclude dead cells. There was no significant difference (ns) in TOROID formation between with (TDP-43) or without (V) TDP-43 expression in the double deletion strain. Live cells with GFP-TOR1 cytoplasmic foci were averaged from 3 independent transformants by examining about 500 cells per transformant. Error bars are SEMs from three independent transformants. Asterisks (***) indicate p < 0.001 in paired two-tailed t-tests, ns indicates no significant difference.

Previously described modifiers that reduce TDP-43’s inhibition of autophagy were also found to lessen TDP-43’s inhibition of TOROID formation, measured by the appearance of TOR1-GFP foci 10. Likewise, we find that *apt1*Δ* aah1*Δ partially restores TOROID formation in cells expressing TDP-43 (**Fig. 3**).

### Examining the effect of *apt1*Δ* aah1*Δ on the nature of TDP-43 foci 

**Figure 4:**
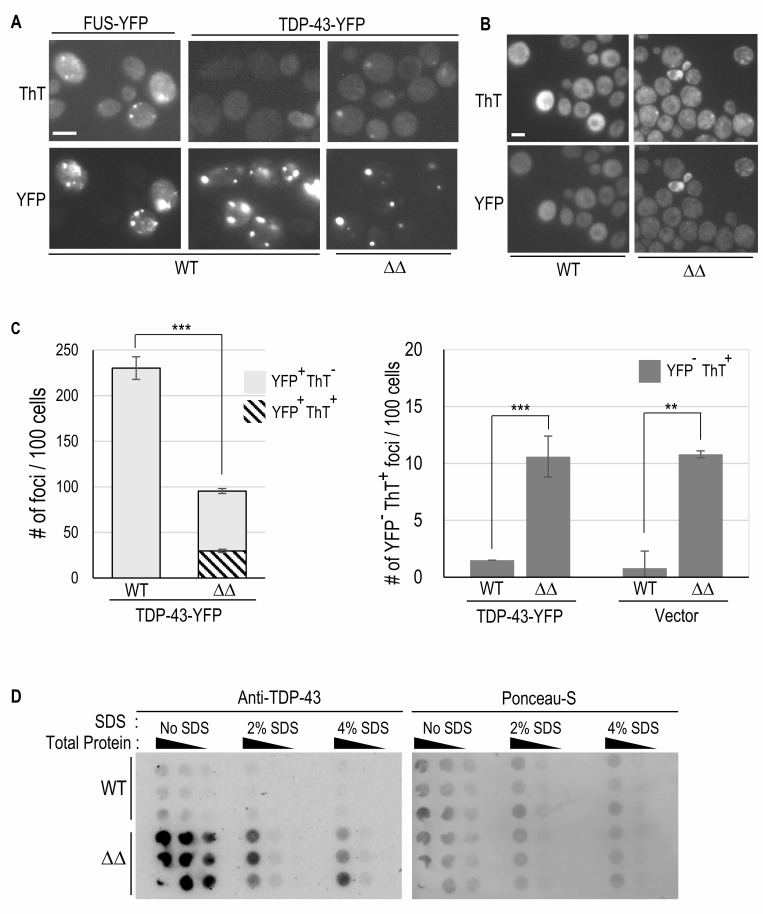
FIGURE 4: The double *aah1*Δ *apt1*Δ deletion makes TDP-43 aggregates more amyloid-like. **(A) **TDP-43 forms thioflavin-T foci in *aah1*Δ *apt1*Δ deletion strains**.** BY4741 (WT) and isogenic double deletion strains (ΔΔ) were transformed with *YCpGAL-TDP43-YFP-U* or *YCpGAL-FUS-YFP-U*. Cells grown on selective galactose plates lacking adenine and expressing TDP-43-YFP or FUS-YFP were collected, stained with Thioflavin-T (ThT), and examined for CFP fluorescence to see the ThT staining (Top), and YFP fluorescence (Bottom). **(B)** Thioflavin-T foci formed in *aah1*Δ *apt1*Δ deletion strains in the absence of TDP-43. BY4741 (WT) and* aah1*Δ *apt1*Δ deletion strains were transformed with *pYCpGAL-YFP-U*. Cells expressing YFP on selective galactose plates lacking adenine were collected, stained, and examined as in (A). Scale bar is 10 µm. **(C)** Quantification of TDP-43-YFP (YFP^+^) and thioflavin T foci (ThT^+^) or non-thioflavin foci (ThT^-^) presence and overlap. Number of foci per 100 cells in WT and double* aah1*Δ *apt1*Δ deletion (ΔΔ) strains transformed withTDP-43-YFP or empty vector are shown. Foci are detected via YFP fluorescence or thioflavin T (ThT) staining (CPF fluorescence). Left counts only YFP^+ ^foci in 100 cells exhibiting fluorescence (either diffuse or foci). In the *aah1*Δ *apt1*Δ deletion (ΔΔ) strain, 30% of YFP^+ ^foci were ThT^+^. Right shows only YFP^- ^ThT^+^ foci in WT and ΔΔ transformed with either TDP-43-YFP or vector. Statistical significance was determined with a two-tailed t-test (** = p <0.01 and *** = p<0.001). **(D)** Filter trap assay shows SDS-resistant aggregation of TDP-43-YFP in an* aah1*Δ *apt1*Δ deletion (ΔΔ) yeast strain. Lysates of yeast expressing TDP-43-YFP in either WT or ΔΔ strains were heated to 95°C for 7 minutes with or without SDS (0%, 2%, or 4%). They were then subjected to a filter trap assay, followed by immunodetection with an anti-TDP-43 antibody (left panel), stripping and staining with Ponceau-S (right).

The frequency of TDP-43-YFP foci was higher (230 foci per 100 cells) in a wild-type vs. its isogenic *apt1*Δ* aah1*Δ derivative (95 foci per 100 cells) (**Figs. 4A, C**). In wild-type strains TDP-43-YFP usually forms one large irregularly shaped focus as well as multiple small foci. In these cells no foci are detected with thioflavin T staining. In the *apt1*Δ* aah1*Δ derivative there were seldom any small foci and the larger foci present were more punctate and slightly smaller than the large foci in wild-type cells (**Figs. 1B** and **4A**). Surprisingly, many (~30%) of the TDP-43-YFP foci in the *apt1*Δ* aah1*Δ strain also stained with thioflavin T (**Fig. 4A**, **C** left). Although these thioflavin T^+^ TDP-43-YFP foci are not as bright as those seen for FUS-YFP foci, they are clearly visible. Also, while most (75%) of the thioflavin T foci that appear in the *apt1*Δ* aah1*Δ cells expressing TDP-43-YFP are also visible in the YFP channel, about 25% are not. Indeed, a similar frequency of thioflavin T foci was also seen in *apt1*Δ* aah1*Δ cells not expressing TDP-43 (**Fig. 4B, C** right). These are likely amyloid adenine foci as previously described in *apt1*Δ* aah1*Δ cells 32. Essentially no thioflavin T foci were seen in wild-type cells lacking TDP-43 and FUS (**Fig. 4B, C** right)

Further evidence that TDP-43 aggregates become amyloid in the *apt1*Δ* aah1*Δ strain is our finding that TDP-43 aggregates are resistant to boiling in the presence of SDS (Sodium Dodecyl Sulfate) in the *apt1*Δ* aah1*Δ but not wild-type strain (**Fig. 4D**). We used a filter trap assay that captures protein aggregates on a PVDF (polyvinylidene difluoride) membrane, allowing detection of SDS-resistant protein aggregates. TDP-43 aggregates formed in the *apt1*Δ* aah1*Δ strain, but not the wild-type strain, were retained on the membrane indicating increased SDS-resistant aggregation. Ponceau-S staining (right panel) confirms total protein loading. These results suggest that the ΔΔ stabilizes TDP-43-YFP aggregates, rendering them less susceptible to SDS solubilization which is characteristic of amyloids.

**Figure 5:**
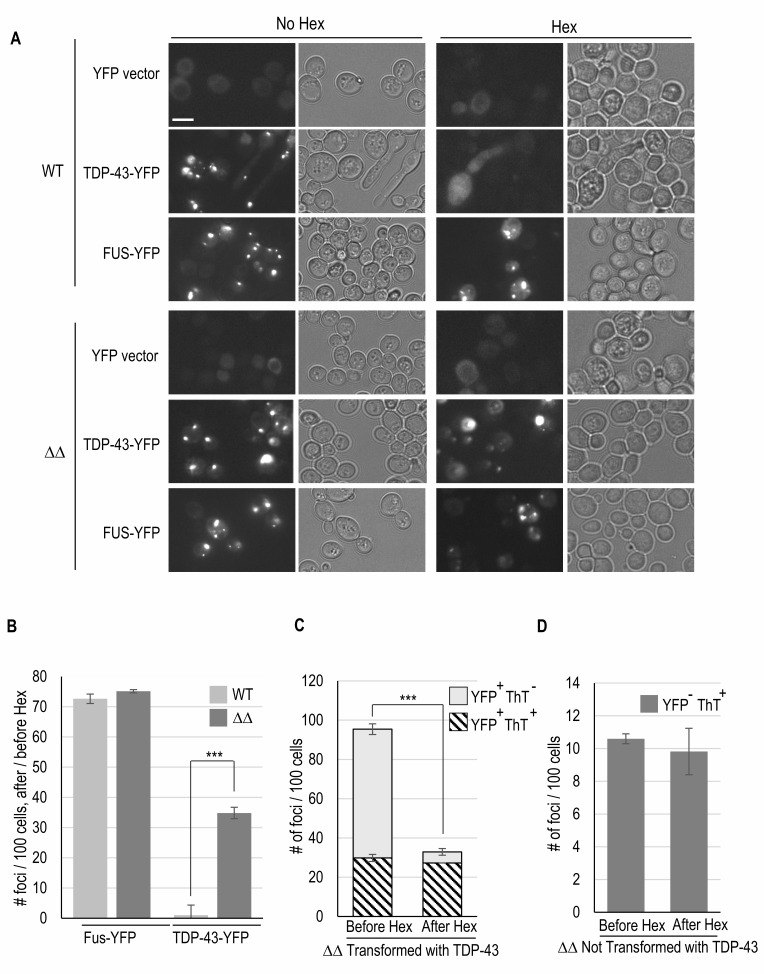
FIGURE 5: The double *aah1*Δ* apt1*Δ deletion strain makes TDP-43 aggregates less liquid-like. Isogenic wild-type (WT) and *aah1*Δ* apt1*Δ deletion strains transformed with plasmid expressing YFP (YFP vector), TDP-43-YFP or FUS-YFP were grown overnight in plasmid selective galactose media lacking adenine. Their YFP fluorescence was examined prior to (No Hex) and following treatment with 10% 1,6-hexanadiol (Hex) for 5 minutes that dissolves liquid-like aggregates. Three transformants each were tested and gave similar results. **(A)** Hexanediol doesn’t dissolve all the TDP-43-YFP foci in the double* aah1*Δ* apt1*Δ deletion cells. Fluorescent pictures of cells under the microscope were taken with 40ms exposure, bright field images are shown to the right. Scale bar is 10 µm. **(B)** Quantification of foci in (A). ** indicates p <0.001 in a paired two-tailed t-test. The absence of stars indicates no statistical difference. **(C)** Hexanediol doesn’t dissolve foci that stain with thioflavin T in the *aah1*Δ* apt1*Δ strain. TDP-43-YFP and thioflavin T foci presence and overlap were quantified before and after treatment with hexanediol. In ΔΔ cells transformed with *YCpGAL-TDP43-YFP-U* the number of YFP fluorescent foci (YFP^+^) per 100 cells that do (ThT^+^) or do not (ThT^-^) stain with thioflavin T are shown before and after treatment with hexanediol. **(D)** ΔΔ cells not transformed with TDP-43-YFP, thioflavin T foci (YFP^-^ ThT^+^) before and after cells were treated with hexanediol were counted.

While the *apt1*Δ* aah1*Δ double deletion makes TDP-43-YFP more amyloid-like, it also reduces the liquid -like droplet nature of the TDP-43-YFP foci. This was evident because in the *apt1*Δ* aah1*Δ strain, cells with TDP-43-YFP foci retained foci following hexanediol treatment (**Fig. 5A, B**). In contrast, in wild-type cells TDP-43-YFP foci were all dissolved by hexanediol, indicating that they are liquid-like droplets rather than amyloid 10. FUS-YFP foci were used as controls because they are amyloid. These foci stain with thioflavin T and are not dissolved by hexanediol 10. Furthermore, when *apt1*Δ* aah1*Δ cells with TDP-43-YFP foci were treated with hexanediol, 90% of the remaining TDP-43-YFP foci stained with thioflavin T (**Fig. 5C**). This is consistent with the idea that hexanediol dissolves the liquid-like foci leaving amyloid foci intact. Likewise, hexanediol did not reduce the frequency of thioflavin T foci stained in *apt1*Δ* aah1*Δ cells not expressing TDP-43-YFP (**Fig. 5D**) supporting the assumption that these foci are amyloid.

**Figure 6:**
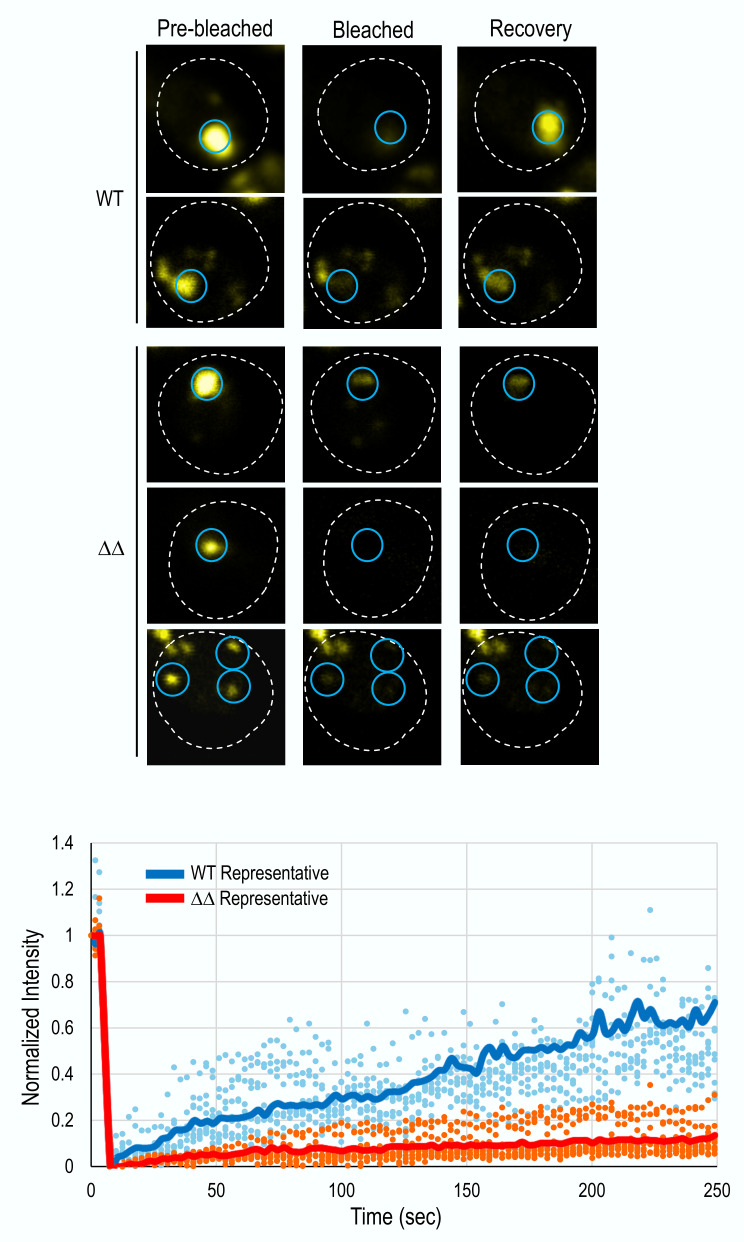
FIGURE 6: FRAP analysis of TDP-43-YFP foci in wild-type and *aah1*Δ* apt1*Δ deletion strains. BY4741 (WT) and double deletion (ΔΔ) strains were transformed with *pGAL-TDP43-YFP-U* and grown in 2% galactose media with 1% raffinose to induce TDP-43-YFP expression. FRAP (fluorescence recovery after photobleaching) was performed on individual TDP-43-YFP foci to assess their dynamic behavior. **Top:** Representative images of FRAP. For each strain, selected foci (blue circles) were photobleached, and fluorescence recovery was monitored over time. Dotted white lines outline the cell boundary. WT cells display visible fluorescence recovery after bleaching, whereas ΔΔ cells show minimal to no recovery, indicating reduced mobility of molecules in TDP-43-YFP foci. **Bottom:** Quantification of normalized fluorescence intensity over time for individual foci (WT in blue, ΔΔ in red). Each dot represents a single time point from a single focus (n = 9 for the WT strain; n=11 for the (( strain). Solid lines represent the average fluorescence recovery curves for each strain (WT: blue line, ΔΔ: red line), serving as representative traces. Similar data was obtained on a different day when 11 foci in WT cells and 6 foci in ΔΔ cells were examined (see supplementary data).

Finally, fluorescence recovery after photobleaching (FRAP) showed that TDP-43-YFP foci formed in wild-type cells recovered much more efficiently than those in *apt1*Δ* aah1*Δ cells (**Fig. 6 **and Supplementary data). Wild-type foci gradually recovered, indicating dynamic and mobile assemblies, while foci in ΔΔ remained mostly immobile, suggesting they are amyloid.

## DISCUSSION

Previous studies have shown that the ability of amyloids to influence the aggregation of heterologous proteins, either promoting or inhibiting amyloid formation and by altering the type (strain) of amyloid promoted 21,22,23,24,25,26,27. In this study, we aimed to determine whether adenine fibers could affect the aggregation and toxicity of TDP-43 in yeast.

The results indicate that the accumulation of adenine, and the formation of adenine amyloid-like fibers, significantly affects the aggregation properties and toxicity of TDP-43 in yeast. The double deletion of *APT1* and *AAH1,* previously shown 32 to cause the accumulation of adenine amyloid-like fibers, reduces the toxicity of TDP-43 and the ability of TDP-43 to elongate cells. This occurs without lowering the levels of TDP-43 protein in the cell. Interestingly, these effects are not further enhanced by increasing adenine levels, which is surprising given that elevated adenine increases amyloid-like adenine fiber formation 32. This lack of additional effect may be due to adenine itself being toxic at higher concentrations in the double deletion strain, thus masking any beneficial impact on TDP-43 toxicity. Like other modifiers of TDP-43 toxicity 10,20, the double deletion reduces the inhibition of autophagy and TOROID formation caused by expression of TDP-43. This reduction occurs even though the double deletion itself does not affect autophagy or TOROID formation in the absence of TDP-43.

The TDP-43 foci formed in the double deletion strain differ from those in wild-type cells. All 20 TDP-43 foci tested for FRAP in the double deletion showed reduced mobility compared to the 17 TDP-43 foci tested in wild-type cells. Furthermore, while all TDP-43 foci in wild-type cells are dissolved by hexanediol, 30% of the TDP-43 foci in the double deletion are resistant to hexanediol and 90% of these stain with thioflavin T. Therefore, TDP-43 foci in the double deletion are less-liquid like and more amyloid like. These amyloid-like foci appear to be less toxic and less disruptive to autophagy and TOROID formation than the liquid-like TDP-43 foci formed in wild-type cells. Importantly, the data indicate that liquid-like TDP-43 foci are the toxic species. The FRAP results indicate that all of the TDP-43 foci in the double deletion strain have become more amyloid like even though they aren’t all stained with thioflavin T and some can still be dissolved by hexanediol.

One possible explanation for these differences is cross-seeding between adenine fibers and TDP-43. Cross-seeding is a well-established phenomenon where aggregates of one amyloid protein can influence the aggregation of another. *In vitro *studies have shown that hnRNPA1 hydrogels can directly cross-seed the aggregation of FUS, another amyloid-forming protein 36. It is plausible that a similar mechanism is at play here, where adenine fibers interact with TDP-43, altering its aggregation state and reducing its toxicity.

This hypothesis raises an interesting question: if adenine fibers are indeed interacting with TDP-43, why does the double deletion strain reduce TDP-43 toxicity even without added adenine? This could be explained by the fact that the double deletion induces a baseline level of intracellular adenine amyloid-like fiber, even without added adenine 32. This low level of fibers might be sufficient to affect TDP-43 aggregation, so any additional adenine beyond this threshold might not further influence the properties of TDP-43 aggregates.

While the observed effects might be indirect, the ability of excess cellular adenine to influence TDP-43 aggregation properties supports the idea that amyloid-like adenine fibers can affect other cellular amyloids or aggregates. This suggests a broad role for metabolite-based amyloid structures in modulating the behavior of pathological protein aggregates in cells.

## CONCLUSION

In summary, the paper shows that the double deletion of *APT1* and *AAH1* reduces TDP-43’s toxicity and inhibition of autophagy and TOROID formation. At the same time, the double deletion makes TDP-43 foci more amyloid like as judged by reduced mobility measured with FRAP, an increase in thioflavin T staining, and reduced dissolution by hexanediol. Possibly cross-seeding TDP-43 with adenine fibers, results in the altered TDP-43 aggregate properties. These results open new avenues for exploring the role of metabolite-based amyloids in modulating protein aggregation and toxicity, with potential implications for understanding the broader landscape of protein misfolding diseases. Further studies will be required to determine whether these effects are mediated through direct interactions between adenine fibers and TDP-43.

## MATERIAL AND METHODS

### Strains and plasmids 

Strains used are shown in **Table 1**, plasmids in **Table **2. To construct the *apt1*Δ* aah1*Δ strain, we began with BY4741 37 containing *apt1:: hphMX6 *obtained from Matin Kupiac’s collection via Dana Laor 32 and BY4741 *aah1::KanMX6 *from the deletion library 38. We transformed the BY4741 *apt1::hphMX6 *strain with a PCR-amplified* aah1::KanMX6 *fragment using upstream and downstream gene-specific 45mer primers. Cells were first grown on a YPD plate before being plated on YPD containing 200 μg /ml G418 to select for double deletion strains. These strains were further validated by growth on YPD containing 200 μg /ml hygromycin. The gene disruptions were confirmed with PCR.

**Table 1:** Strains.

**Strains**	**Description**	**Reference**
BY4741	*MAT***a** *his3*Δ*1 leu2*Δ*0 met15*Δ*0 ura3*Δ*0*	38
*apt1*Δ	BY4741 *apt1::hphMX6*	32
*aah1*Δ	BY4741 *aah1::KanMX6*	Open Biosystems (Huntsville, AL, USA)
*apt1*Δ* aah1*Δ	BY4741 *apt1::hphMX6*, *aah1::KanMX6*	This study

**Table 2:** Plasmids.

**Short name, SWL laboratory plasmid #**	**Description**	**Reference**
pSK108	pRS305--PTOR1 (1000 bases)-GFP-TOR1 (426 N-terminal bases), LEU2	39
YCpGAL-EYFP, p2260	pAG416 Gal-ccdB–EYFP (*URA3, CEN*)	Addgene plasmid #14219, made by Aaron Gitler
YCpGAL-TDP43YFP, p2042	pRS416 Gal-TDP YCpGAL-TDP-43-YFP, (*URA3, CEN*)	Addgene plasmid #27447, deposited by Aaron Gitler
YCpGAL-FUS-YFP, p2043	pRS416 Gal-FUS-EYFP (*URA3, CEN*)	14
YCpGAL-U, p2129	pAG416 Gal-ccdB (*URA3, CEN*)	Addgene plasmid #14147, made by Aaron Gitler
YCpGAL-TDP43-U, p2665	pAG416 Gal-TDP-43 (*URA3, CEN*)	10
YCpGAL-L, p2245	pAG415 Gal-ccdB (*LEU2, CEN*)	Addgene plasmid #14145, made by Aaron Gitler
YCpGAL-TDP43-L, p2368	pAG415 Gal-TDP-43 (*LEU2, CEN*)	10
YCpCUP1-GFP-ATG8, p2571	pCUP1-GFP-ATG8 (*URA3, CEN*)	Addgene #49423, deposited by Daniel Klionsky

To measure TOROID formation, we disrupted *TOR1* while integrating *GFP-TOR1* controlled by its native promoter into BY4741. Wild-type BY4741 or *apt1*Δ* aah1*Δ strains were transformed with *Spe*I-digested integrating pSK108 plasmid (*LEU2*) containing the TOR1 promoter and GFP tagged N-terminal region (kindly sent by Dr. Noda) 39 and selecting for transformants on leucine-less media. *Spe*l makes a unique cut in the N-terminal *TOR1* region on the plasmid. The correct insertions were confirmed with PCR. Plasmid pSK108 is described in **Table 2**.

### Scoring for growth

Standard yeast medium was used throughout 40. To assess the growth of *apt1*Δ* aah1*Δ cells expressing plasmids, transformants were grown overnight on plasmid selective synthetic glucose medium lacking adenine. Cells to be examined for growth on TDP-43 expressing galactose plates were then normalized to OD_600_ = 4, serially diluted in water 10X, spotted on synthetic medium plates made with 2% glucose or 2% galactose supplemented with required amino acids, yeast nitrogen base without amino acids, and 0 or 1 mg/L adenine, and incubated at 30°C for four days. Cells to be used for growth curves were normalized to OD_600_= 0.2 in synthetic raffinose/galactose (SRafGal) medium lacking adenine. A total of 200 µL of the diluted cultures was transferred into a 96-well plate and incubated at 30°C for 25 hours with continuous shaking. OD_600_ readings were recorded using a SpectraMax M5 (Molecular Devices, Sunnyvale, CA) microplate reader. The data presented are representative of experiments performed in triplicate.

### Microscopy for YFP tagged proteins before and after 1,6 hexanediol treatment, and for thioflavin T-stained protein 

A Nikon Eclipse E600 fluorescent microscope (Nikon, Tokyo, Japan) with 100×/1.23 NA or 60×/1.4 NA oil immersion objectives was used to observe YFP fluorescence with a YFP filter, with or without prior 1,6 hexanediol treatment. Cells were also observed in brightfield. Thioflavin T-stained fluorescent foci were observed using a CFP filter. Post-acquisition image processing (brightness and contrast) was performed using Adobe Photoshop 2024 (San Jose, California), and all micrographs display a scale bar. Detailed methods have been described previously 10.

### Scoring for autophagy by determination of GFP-ATG8 cleavage with western blots and microscopic fluorescence counting 

BY4741 WT and its isogenic double *apt1*Δ* aah1*Delta; deletion strain were doubly transformed with YC*pGAL-TDP-43-L* and YC*pCUP1-GFP-ATG8*. The transformants grown on plasmid selective glucose plates lacking adenine were normalized to 0.2 OD_600_ and spread on TDP-43 and GFP-ATG8 overexpressing plasmid selective galactose plates with 1% raffinose and 50 µM CuSO_4_. Preparation of lysates and immunoblotting were as described previously 20. α-GFP (1:5000, Roche); α-TDP-43 (1:3000; Proteintech Group); and α-PGK (yeast 3-phosphoglycerate kinase, 1:10,000, Novex) were respectively used to compare the levels of uncleaved GFP-ATG8 and cleaved GFP, TDP-43, and PGK (yeast 3-phosphoglycerate kinase). The ratio of cleaved GFP to uncleaved GFP-ATG8 measures autophagy. The internal PGK control was used to compare the level of TDP-43 present in the wild-type (WT) vs. the double deletion strain (ΔΔ).

Microscopic determination of the cellular location of GFP-ATG8 was also used to measure autophagy. Autophagy was calculated as the fraction of cells with either no fluorescence or with fluorescence only in the vacuole over total live cells. Dead cells were detected with trypan blue staining seen in brightfield. Microscopic determination of the location of the GFP-ATG8 fluorescence was as previously reported 20.

### Measuring TOROID formation 

TOROID formation was measured as described previously 20.

### Filter trap assay

The filter trap assay was done as described previously 41,42. Briefly, yeast cell lysates expressing TDP-43-YFP were prepared in buffer B (50 mM Tris-HCl, pH 7.0, 150 mM NaCl) and heated at 95°C for 7 minutes in the presence or absence of SDS (0%, 2%, or 4%). Samples were then subjected to vacuum filtration through a PVDF membrane using a 96-well dot blot apparatus (Bio-Rad Laboratories, Hercules, CA). After filtration, membranes were washed twice with the respective SDS concentration used in sample preparation and blocked in 2% Tropix O-Block (Applied Biosystems, Bedford, MA) in blocking buffer (100 mM Tris-HCl, pH 7.4, 150 mM NaCl) for 1 hour. Membranes were incubated with primary antibodies against TDP-43 at a 1:2000 dilution for 1 hour. After washing twice with blocking buffer, membranes were incubated with AP-conjugated secondary antibody for 1 hour. Following additional washes, the membranes were developed using the Tropix CDP-Star System (Applied Biosystems, Bedford, MA). The membrane was then stripped and stained with Ponceau-S to quantify total protein present.

### Fluorescence recovery after photobleaching (FRAP)

FRAP assays were performed as previously described 11, with minor modifications. Briefly, yeast cells expressing TDP-43-YFP in either wild-type or double deletion (ΔΔ) strains were cultured overnight in selective galactose medium supplemented with 1% raffinose to induce protein expression. Cells were immobilized onto microscopy slides pre-coated with 1 mg/mL concanavalin A (Sigma-Aldrich, St. Louis and Burlington, MA) to promote cell adhesion. FRAP experiments were conducted using a Leica Stellaris 8 confocal microscope (Leica Microsystems, Germany) equipped with an 86× water-immersion objective. Image acquisition was performed at a resolution of 512 × 512 pixels. Targeted photobleaching of defined TDP-43-YFP foci was performed using a white laser set at 513 nm and 80% laser intensity. For each sample, approximately 120 frames were acquired including 5 pre-bleach frames at 1.3-second intervals, 15 bleach frames at 0.3-second intervals, and subsequent recovery imaging for up to 250 seconds at 1.3-second intervals. Fluorescence intensity within the region of interest (ROI) was measured using LAS X software (Leica Microsystems, Germany), normalized to the pre-bleach signal, and recovery curves were analyzed as previously described 43.

## CONFLICT OF INTEREST

The authors declare no conflict of interest.

## SUPPLEMENTAL MATERIAL

Click here for supplemental data file.

All supplemental data for this article are available online at www.microbialcell.com/researcharticles/2025a-park-microbial-cell/.
