# Supplementary material for: An adenine model of inborn metabolism errors alters TDP-43 aggregation and reduces its toxicity in yeast revealing insights into protein misfolding diseases
Source: Microb Cell. 2025 May 22;12:119–30. doi: 10.15698/mic2025.05.850 (PMC12105045; doi:10.15698/mic2025.05.850)
Supplement: Supplementary file 1 [file mic-12-119-s01.pdf]

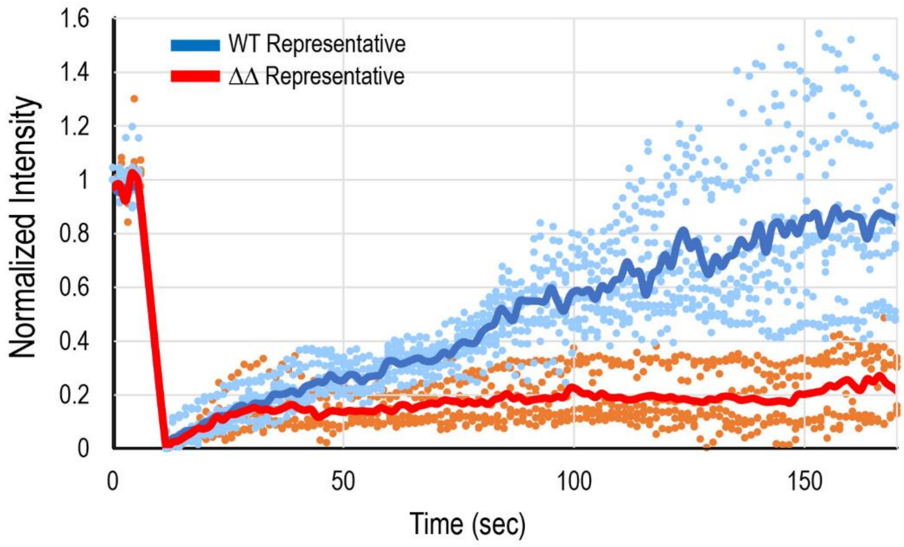


**FRAP analysis of TDP-43-YFP foci in wild-type and *aah1Δ apt1Δ* deletion strains.** As in Figure 6, BY4741 (WT) and double deletion (ΔΔ) strains were transformed with *pGAL-TDP43-YFP-U* and grown in 2% galactose media with 1% raffinose to induce TDP-43-YFP expression. FRAP (fluorescence recovery after photobleaching) was performed on individual TDP-43-YFP foci to assess their dynamic behavior. Quantification of normalized fluorescence intensity over time for individual foci (WT in blue, ΔΔ in red) is shown. Each dot represents a single time point from a single focus (n = 11 for the WT strain; n=6 for the ΔΔ strain). Solid lines represent the average fluorescence recovery curves for each strain (WT: blue line, ΔΔ: red line), serving as representative traces.
